# Successful Treatment by Surgery and Lenvatinib of a Patient with Adrenal Metastasis of Papillary Thyroid Cancer

**DOI:** 10.1155/2020/2107430

**Published:** 2020-11-05

**Authors:** Hajime Nakamura, Kohichi Takada, Kazuyuki Murase, Hiroki Sakamoto, Naotaka Hayasaka, Kazuma Ishikawa, Yuki Ikeda, Makoto Yoshida, Satoshi Iyama, Ko Kobayashi, Tetsuya Shindo, Shintaro Sugita, Koji Miyanishi, Masayoshi Kobune, Naoya Masumori, Junji Kato

**Affiliations:** ^1^Department of Medical Oncology, Sapporo Medical University School of Medicine, Japan; ^2^Department of Hematology, Sapporo Medical University School of Medicine, Japan; ^3^Department of Urology, Sapporo Medical University School of Medicine, Japan; ^4^Department of Surgical Pathology, Sapporo Medical University School of Medicine, Japan

## Abstract

Papillary thyroid cancer (PTC) is considered an indolent cancer, but some PTC patients do present with distant metastases and treatment strategies for such patients are not well established. Recently, lenvatinib, an inhibitor of multiple tyrosine kinases, has been introduced to treat patients with advanced PTC but carries a risk of serious adverse events such as hemorrhage. Here, we report a PTC patient with a left adrenal metastasis and lenvatinib-induced hemorrhage who underwent successful surgical resection and was subsequently treated with a lower dose of lenvatinib. The patient has now been in a stable state with no adverse events for nearly two years. This case highlights the importance of surgical resection of metastatic PTC and subsequent lenvatinib therapy, even when the tumor is at an advanced stage.

## 1. Introduction

Papillary thyroid cancer (PTC) is the most common type of thyroid cancer. PTC is a differentiated thyroid cancer (DTC) considered to be indolent because distant metastases are seen in only a small minority of patients. Standard therapies for metastatic DTC include treatment with radioactive iodine (RAI) [1], but patients who become refractory to this treatment have a poor prognosis [2]. When PTC does metastasize, the sites most frequently infiltrated are the lung and bone [3]. Adrenal metastases are extremely rare, with only a few cases reported [4]. As a result, no treatment strategies are well established for this condition. Lenvatinib, a novel inhibitor of multiple tyrosine kinases, has been approved for treating patients with radioiodine-refractory- (RR-) DTC. Lenvatinib can dramatically suppress DTC but may evoke lethal adverse events such as bleeding [5].

Here, we present a patient with RR-PTC successfully treated by resection of a left adrenal metastasis despite lenvatinib-induced intratumoral hemorrhage. We were able to continue lenvatinib treatment in this patient at a lower dose with no severe adverse effects.

## 2. Case Presentation

A 70-year-old woman was first diagnosed with PTC at the age of 53. She subsequently underwent a total thyroidectomy together with regional lymph node dissection, followed by iodine-131 (I-131) therapy immediately following the initial diagnosis. The TNM classification at the first pathological diagnosis was T4aN1aM0. Recurrence did not occur until 10 years after the patient's initial treatment when multiple lung and supraclavicular lymph node metastases were identified during late follow-up. The patient received stereotactic radiotherapy (58 Gy/29 Fr) for the lymph node metastasis and a second course of I-131 therapy. However, metastases in both lung and lymph node gradually progressed despite such therapy. The patient underwent a third round of I-131 therapy three years after the second. Since then, these tumors have remained unchanged for almost 5 years, indicating stable disease (SD).

**Figure 1:**
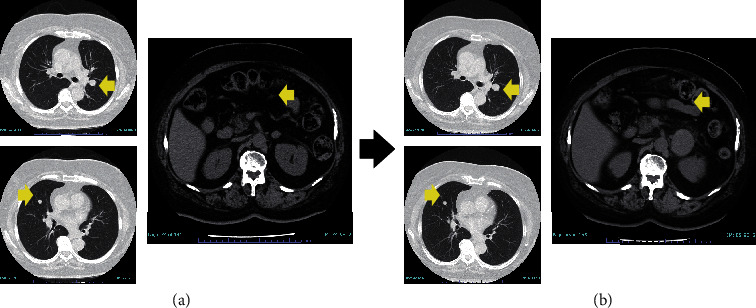
Computed tomography of lung tumors and a left adrenal tumor (arrows). (a) At the time when the left adrenal tumor was initially identified. (b) Six months after the initial diagnosis of the left adrenal tumor.

**Figure 2:**
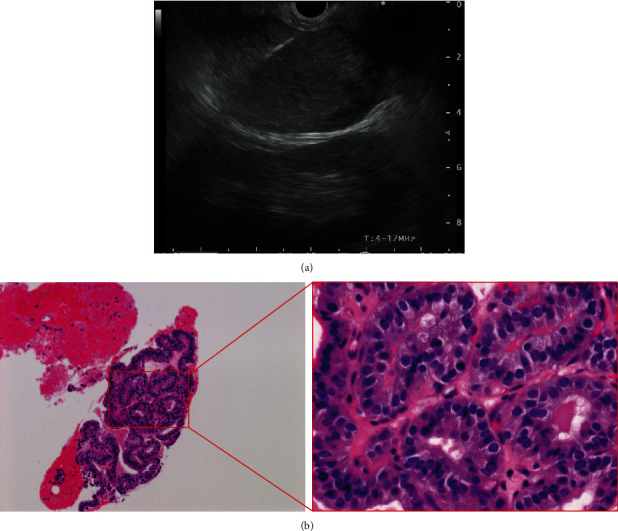
Endoscopic ultrasound-guided fine-needle aspiration (EUS-FNA) procedure and histopathological findings. (a) EUS-FNA was performed on the left adrenal tumor from the posterior wall of the stomach. (b) Histopathological findings showing a papillary structure similar to that of the resected thyroid cancer.

**Figure 3:**
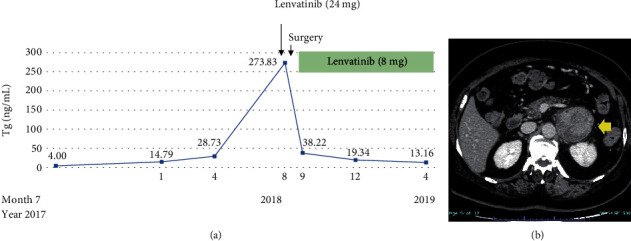
Clinical course of the patient. (a) Serum thyroglobulin level (T) rapidly increased in step with the size of the left adrenal tumor. We initiated 24 mg lenvatinib treatment, but due to uncontrollable intratumoral bleeding, the patient had to undergo surgery. The serum thyroglobulin level immediately decreased after surgery, and the patient was again treated with lenvatinib, now at only 8 mg. (b) Computed tomography findings of intratumoral bleeding of the left adrenal tumor (arrow).

However, the patient presented with a left adrenal tumor 17 years after her initial treatment for PTC. This grew rapidly over only six months, whereas the lung and supraclavicular lymph node metastases remained stable. We initially considered that the adrenal tumor might not have been a PTC metastasis (Figure 1), but the serum thyroglobulin level rapidly increased as high as 273.83 ng/mL in step with the growth of the tumor (although it had previously remained within the normal range (5.0-30.0 ng/mL) since the first diagnosis). We therefore performed an endoscopic ultrasound-guided fine-needle aspiration (EUS-FNA) for the purpose of histopathological assessment. This revealed that the structure of the tumor was similar to that of the resected PTC. Based on these findings, it was concluded that the left adrenal tumor was indeed a PTC metastasis (Figure 2). Immediately after the EUS-FNA, the patient experienced an intratumoral hemorrhage but recovered quickly with supportive care only. We started 24 mg lenvatinib treatment for advanced RR-PTC five days after the EUS-FNA procedure, but 12 h later, the patient experienced sudden back pain due to intratumoral rebleeding (Figure 3). Although lenvatinib is a promising drug against advanced RR-PTC, it is well-recognized that it carries a risk of adverse bleeding events. To prevent further bleeding and to enable continued lenvatinib treatment, we surgically resected the left adrenal metastasis. Subsequently, the serum thyroglobulin level decreased to within the normal range. We retreated with 8 mg lenvatinib, which has now maintained SD without any serious adverse events for almost two years.

## 3. Discussion

According to a previous report, distant metastases from PTC are rare, with only 71 of 5,969 resected PTC cases presenting with disease spread [6]. Of these 71 cases, the most frequently affected site was the lung (60 cases) followed by the bone (5 cases). No adrenal metastases were reported in such patients. The most common systemic modality used to treat patients with metastatic DTC is RAI. The survival rate in patients with I-131 uptake was reported to be 56% at 10 years, whereas in patients without I-131 uptake, it was only 10% at that time. The treatment strategy for such patients is not well established [2].

Several multitarget kinase inhibitor drugs have been introduced recently for the treatment of RR-DTC. Sorafenib was first approved for this indication in 2013 after a phase 3 trial showed significant improvement in progression-free survival (PFS) compared to placebo [7]. Lenvatinib treatment also resulted in a significant prolongation of PFS and an improved response rate among patients with RR-DTC [8]. Vandetanib treatment also yielded a significant prolongation of PFS in a phase 3 trial of patients with advanced medullary thyroid carcinoma [9]. Thus, treatment options for RR-DTC are increasing owing to the development of such drugs. However, several well-known distinct adverse events are associated with the use of multitarget kinase inhibitors, of which some may even be fatal, such as hemorrhage with lenvatinib therapy.

In the PTC case described here, surgical resection of a left adrenal metastasis after lenvatinib-induced hemorrhage was performed. Reports of PTC cases with surgical resection of distant metastases are very limited, but long-term survival after the resection of a solitary pancreatic metastasis of thyroid cancer has been reported [10]. Our patient experienced severe back pain when hemorrhage occurred, which seriously compromised her quality of life, such that we considered it impossible to continue lenvatinib treatment without a risk of rebleeding. However, after surgical resection, the serum thyroglobulin level immediately dropped, and the patient was treated again with 8 mg lenvatinib, which led to a stable condition without serious events for almost two years. Thus, surgical resection of this distant metastasis almost certainly contributed to the patient's long-term survival.

A dose of 8 mg is lower than the recommended initial dose of lenvatinib for RR-DTC. Another successful instance where adverse events of lenvatinib were managed by individualizing the drug schedule in terms of reduced dose and timing, with a significant dose intensity reduction, has also been reported [11]. Our case highlights the efficacy and improved patient safety of a lower dose of lenvatinib for RR-DTC.

Our findings suggest that surgical treatment is an option for rapidly growing metastatic tumors of PTC origin, even at an advanced stage with multiple lung metastases. If a hemorrhage induced by lenvatinib can be managed satisfactorily, lenvatinib administration at a lower dose may be continued for the treatment of metastatic RR-PTC.
